# Stroke unit care in germany: the german stroke registers study group (ADSR)

**DOI:** 10.1186/s12883-017-0819-0

**Published:** 2017-03-09

**Authors:** Steffi Hillmann, Silke Wiedmann, Viktoria Rücker, Klaus Berger, Darius Nabavi, Ingo Bruder, Hans-Christian Koennecke, Günter Seidel, Björn Misselwitz, Alfred Janssen, Christoph Burmeister, Christine Matthis, Otto Busse, Peter Hermanek, Peter Ulrich Heuschmann, on behalf of the German Stroke Register Study Group (ADSR), M. Eßer, S. Rode, B. Hoffmann, R. Hohnhold, A. Reihs, M. Kalic, S. Dienlin, H. Raspe, P. Kolominsky-Rabas, I. Bruder, S. Dienlin, P. Heuschmann, B. Misselwitz, A. Reihs

**Affiliations:** 1 Institute of Clinical Epidemiology and Biometry (ICE-B) Würzburg, Josef-Schneider-Str. 2 / D7, 97080 Würzburg, Germany; 2 Comprehensive Heart Failure Center, University of Würzburg, Straubmühlweg 2a, 97078 Würzburg, Germany; 3 Quality Assurance Project”Stroke Register Northwest Germany”, Institute of Epidemiology and Social Medicine, University of Münster, Albert-Schweitzer-Campus 1, Gebäude D3, 48149 Münster, Germany; 4 Department of Neurology, Vivantes Klinikum Neukölln, Berlin, Rudower Straße 48, 12351 Berlin, Germany; 5 Office for Quality Assurance in Hospitals (GeQiK) Stuttgart at Baden-Wuerttembergische Hospital Federation, Stuttgart, Birkenwaldstr. 151, 70191 Stuttgart, Germany; 6 Vivantes Klinikum im Friedrichshain, Berlin, Landsberger Allee 49, 10249 Berlin, Germany; 7 Department of Neurology, Asklepios Klinik Nord, Hamburg,, Tangstedter Landstraße 400, 22417 Hamburg, Germany; 8 Institute of Quality Assurance Hesse (GQH), Frankfurter Str. 10, 65760 Eschborn, Germany; 9 Quality Assurance in Stroke Management in North Rhine–Westphalia, Medical Association North Rhine, Tersteegenstr. 9, 40474 Düsseldorf, Germany; 10 Institute of Quality Assurance Rhineland-Palatinate / SQMed, Wilhelm-Theodor-Römheld-Straße 34, 55130 Mainz, Germany; 11 Quality Association for Acute Stroke Treatment Schleswig-Holstein (QugSS), Institute of Social Medicine and Epidemiology, University of Lübeck, Ratzeburger Allee 160, 23538 Lübeck, Germany; 12 German Stroke Society, Berlin, Reinhardtstr. 27C, 10117 Berlin, Germany; 13 Bavarian Permanent Working Party for Quality Assurance, Munich, Westenriederstr. 19, 80331 Munich, Germany; 14 Clinical Trial Center Würzburg, University Hospital Würzburg, Josef-Schneider-Str. 2 / D7, 97080 Würzburg, Germany

**Keywords:** Stroke unit care, Quality of health care, Quality indicators, Stroke register

## Abstract

**Background:**

Factors influencing access to stroke unit (SU) care and data on quality of SU care in Germany are scarce. We investigated characteristics of patients directly admitted to a SU as well as patient-related and structural factors influencing adherence to predefined indicators of quality of acute stroke care across hospitals providing SU care.

**Methods:**

Data were derived from the German Stroke Registers Study Group (ADSR), a voluntary network of 9 regional registers for monitoring quality of acute stroke care in Germany. Multivariable logistic regression analyses were performed to investigate characteristics influencing direct admission to SU. Generalized Linear Mixed Models (GLMM) were used to estimate the influence of structural hospital characteristics (percentage of patients admitted to SU, year of SU-certification, and number of stroke and TIA patients treated per year) on adherence to predefined quality indicators.

**Results:**

In 2012 180,887 patients were treated in 255 hospitals providing certified SU care participating within the ADSR were included in the analysis; of those 82.4% were directly admitted to a SU. Ischemic stroke patients without disturbances of consciousness (*p <* .0001), an interval onset to admission time ≤3 h (*p <* .0001), and weekend admission (*p <* .0001) were more likely to be directly admitted to a SU. A higher proportion of quality indicators within predefined target ranges were achieved in hospitals with a higher proportion of SU admission (*p =* 0.0002). Quality of stroke care could be maintained even if certification was several years ago.

**Conclusions:**

Differences in demographical and clinical characteristics regarding the probability of SU admission were observed. The influence of structural characteristics on adherence to evidence-based quality indicators was low.

**Electronic supplementary material:**

The online version of this article (doi:10.1186/s12883-017-0819-0) contains supplementary material, which is available to authorized users.

## Background

Overwhelming evidence from RCTs showed efficacy of stroke unit (SU) care [1] with data from RCTs being replicated in routine care in different countries and different SU settings [2–5]. In German SUs, specific focus is given to management and treatment of patients in the hyper acute phase, including multimodal monitoring of vital functions, rapid diagnosis, and early initiation of secondary prevention [6]. Independent certification of SUs is performed mainly by the German Stroke Society (DSG) and the German Stroke Foundation (SDSH) [7]. Furthermore, stroke units are certified according to regional criteria of the federal states [8]. However, not all patients treated at hospitals providing SU care were admitted to SU. Available data on SU care in Germany are mainly based on routine data [9, 10] estimating for example that about 56.9% of all ischemic stroke (IS) patients in 2012 were admitted to a hospital with a specialized SU care in Germany [9]. However, routine data do not include detailed clinical relevant information such as stroke severity and comorbidities. Thus, it is currently unclear, if the demographic and clinical characteristics influence direct access of stroke patients to a SU. Furthermore, previous studies did not investigate variations in quality of stroke care by SU structures in Germany. Thus, it is unclear to what extent structural characteristics of the respective hospitals, such as certification procedures or percentage of stroke patients admitted to a SU, might influence quality of care provided. Therefore, we investigated the influence of demographic and clinical characteristics of patients on direct admission to a SU as well as the influence of the structural characteristics on adherence to evidence-based quality indicators among hospitals providing SU care in Germany.

## Methods

### The german stroke registers study group (ADSR)

Data were derived from the German Stroke Registers Study Group (ADSR), a voluntary network of 9 regional registers (Bavaria, Baden-Württemberg, Berlin, Hamburg, Hesse, North Rhine, Northwest Germany, Rhineland-Palatinate, and Schleswig-Holstein), which was founded in 1999 for monitoring quality of acute stroke care in Germany [11]. Data are collected mainly electronically, with predefined completeness and plausibility checks, by the respective quality assurance projects [12]. For example: date of ever treatment must be after the date of admission. Only plausible datasets were evaluated and sent to the data-pooling center. Each regional stroke register sends the complete data set in regular intervals to the data-pooling center (University of Würzburg, 2011-2015) [11]. Participation within a regional register of the ADSR is mandatory for all SUs in Germany certified according to the criteria of the DSG and the SDSH (see below) as well as for all hospitals in several regions [12].

### Stroke unit definition

SUs were certified according to criteria of the German Stroke Society (DSG) and the German Stroke Foundation (SDSH) [13] and supported by the LGA InterCert [8]. SUs certified according to these criteria are distinguished into regional SUs and supra-regional SUs, depending on equipment and expertise [6]. Regional SUs have to treat at least 250 patients per year in at least 4 beds [14]. Supra-regional SUs have further demands on structural and procedural requirements [13, 14]. For example, interventional neuroradiology and neurosurgery and all relevant therapeutic and diagnostic facilities for stroke diagnosis, treatment, and management have to be available [6]. Additionally, a minimum of 450 patients per year treated in at least 6 monitoring beds are essential for certification of supra-regional SUs [14]. The minimum number of stroke patients per year was increased up to at least 500 in 2012 [13]. Beside these criteria, certification procedures by the local government of some federal states are also applied (i.e. Baden-Württemberg).

### Hospital characteristics

Analyses were restricted to hospitals with a certified SU, according to criteria of the DSG, the SDSH or to regional criteria by the federal states that provided complete information regarding the admitting department. Investigated structural characterisis of participating hospitals providing SU care included: percentage of patients directly admitted to a SU (SU/general ward/intensive care unit/other), percentage of patients admitted directly to a SU (<60%, 60-80%, >80%), year of first certification of the respective SU (2000-2002, 2003-2005, 2006-2008, 2009-2012), and number of stroke and Transient Ischemic Attack (TIA) patients treated per year (<250, 250-500, >500-750, >750).

### Quality Indicators

A set of evidence based patient-oriented quality indicators was defined to measure quality of acute stroke care in participating hospitals. A standardized process was initiated for developing a set of evidence based quality indicators between 2003 and 2006 [11]. The process contained a systematic literature review, external independent validation and the conduction of a prospective pilot study [11]. The quality indicator set is updated on a regular basis every 2-3 years [15]. Target values of quality indicators were defined to indicate good quality of stroke care based on distribution within the data, national recommendations and expert consensus by the working group [15]. For the present analysis, the following set of 11 patient-oriented QIs with defined target values were used with definitions published previously [12]: early antithrombotic therapy; antithrombotic therapy – secondary prevention; anticoagulant therapy; brain imaging in patients with suspected strokes; vascular imaging in IS and TIA; screening for dysphagia; early rehabilitation – physiotherapy/occupational therapy; early rehabilitation – speech therapy; early mobilization; patients who arrived at the hospital within two hours from symptom onset and received brain imaging within one hour; early systemic thrombolytic therapy in eligible patients.

### Achievement of quality indicators

Achievement of QIs was defined by reaching previously defined target values [12]. The proportion of achieved QIs was calculated by the number of QIs fulfilled in patients divided by the number of QIs that a patient could have fulfilled, according to the defined eligibility criteria of the respective QI.

### Statistical analysis

The following regional stroke audits were included in the present analyses: Hesse, Bavaria, Northwest Germany, Baden-Württemberg, North Rhine and the Berlin Stroke Register. Three regional stroke audits (Hamburg, Rhineland-Palatinate, and Schleswig-Holstein) were excluded because of missing or not comparable information on SU admission. Detailed descriptive and univariate analyses were performed to describe the demographic and clinical characteristics of the patients. Multivariable logistic regression analyses were performed to estimate the characteristics of ischemic stroke patients being admitted to a SU including: age (age group: ≤64y, ≥65y and ≤74y, ≥75y and ≤84y, ≥85y); sex; comorbidities (atrial fibrillation, diabetes mellitus, hypertension, previous stroke, hyperlipidemia); time from onset to admission (≤2h, >2-3h, >3-6h, >6-24h, >24h-48h, >48h, unknown); NIHSS (≤3, >4-15, ≥15) [16]; and day of admission (weekday, weekend). Analyses were adjusted for regional audits. The association between hospital characteristics and the achievement of given target values to a defined set of evidence based quality indicators was assessed by Generalized Linear Mixed Models (GLMM). GLMM was used to estimate the p-values and regression coefficients for the differences in the percentage of fulfilled quality indicators regarding the structure of the hospital (percentage of patients being admitted to a SU, year of certification of the respective SU, and the number of stroke and TIA patients treated per year) and individual characteristics (age [categories], sex, stroke classification [ischemic stroke (IS), intracerebral hemorrhage (ICH), Transient Ischemic Attack (TIA), other], day of admission [weekday/weekend], and NIHSS on admission [categories]). The variable hospital was added as a random effect to model the correlations within the hospitals. Analyses were restricted to patients without missing values in the respective variables. The number of missing values ranged from 0.1% in age to 3.8% in NIHSS. Regarding the variables “interval onset to admission” and “day of admission”, no comparable information was provided in one regional stroke register. Therefore, we performed a sensitivity analysis for the multivariable logistic regression analysis including all patients with missing information as own category. Statistical significance was determined at an alpha level of 0.05. Statistical analyses were performed using the SAS 9.3 Software Package, SAS Institute Inc., Cary, NC, USA.

### Ethics

The data pooling was approved by the ethics committee of the Charité-Universitätsmedizin Berlin (EA4/043/10) and is registered by the University of Würzburg (214/11). The identity of the individual patients was completely anonymous. Therefore, no specific informed consent was obtained by patients. The investigators who performed the data analyzes were blinded to hospital identities. These identities are only known by the coordinating center of the respective regional stroke registers [11].

## Results

In 2012,180,887 patients were included in the analysis from 255 hospitals providing certified SU care: of those, 149,001 patients have been directly admitted to SUs (82.4%) The range of SU admission between the regional stroke audits was 77.9% - 84.6%. Median age of all patients was 74 years (IQR 64-82), 49.1% were women. 66.4% had an (IS), 6.3% had an intracerebral hemorrhage (ICH), 25.9% had a transient ischemic attack (TIA), and 1.4% of strokes were undefined or subarachnoid hemorrhage. Demographical and clinical characteristics of all patients treated in hospitals with a certified SU by direct SU admission are provided in Table 1. The proportion of patients receiving thrombolysis therapy without admission on a SU was relatively high (10.9%). This was due to the high number of thrombolysis therapy at Intensive Care Units (data not shown).

**Table 1:** Demographic and clinical characteristics of the patients

	Hospital with certified SU	Admission on a SU		*P*-value
		Yes	No	
No of centers	255			
No of patients, n	180,887	149,001	31,886	
Age, y				<.0001
Mean (SD)	72.0 (13.4)	72.1 (13.3)	71.6 (14.0)	
Median (IQR)	74 (64 – 82)	74 (64 – 82)	74 (63 – 82)	
Age categories, y, %				<.0001
< 65	25.8	25.4	27.3	
65-74	24.6	24.8	23.9	
75-84	32.6	32.8	31.5	
≥ 85	17.0	16.9	17.3	
Stroke subtype, %				<.0001
IS	66.4	67.5	61.5	
ICH	6.3	4.9	13.0	
TIA	25.9	26.7	22.2	
other (unclassified, SAH)	1.4	0.9	3.3	
Women, %	49.1	48.9	50.4	<.0001
Comorbidities, %				
Atrial fibrillation	25.8	26.0	24.7	<.0001
Diabetes mellitus	26.5	26.8	25.2	<.0001
Hypertension	82.6	83.2	79.5	<.0001
Previous stroke	24.6	24.9	23.1	<.0001
Hyperlipidemia	51.1	52.8	43.4	<.0001
Thrombolysis after IS, %	14.6	15.3	10.9	<.0001
Interval onset admission, hours, %^a^				<.0001
< =2	20.7	21.7	15.9	
> 2-3	9.2	9.7	6.9	
> 3-6	15.3	16.0	11.9	
> 6-24	16.2	17.1	12.2	
> 24-48	5.7	5.7	5.6	
> 48	9.2	7.0	19.8	
unknown	23.8	22.9	27.7	
Neurological signs within 24h after admission, %				
Paresis	55.3	55.9	52.2	<.0001
Aphasia	27.5	27.4	28.2	0.0065
Dysarthria	34.6	35.2	31.5	<.0001
Disturbed level of consciousness	9.0	6.9	18.6	<.0001
NIHSS on admission,				
Median (IQR)	3 (1-7)	3 (1-7)	3 (1-10)	<.0001
NIHSS on admission, %				<.0001
< 4	55.5	55.6	55.1	
4-15	35.0	36.4	27.9	
> 15	9.5	8.0	17.0	
Day of admission				<.0001
Weekday	73.7	73.1	76.3	
Weekend	26.3	26.9	23.7	
Length of stay, days				<.0001
Mean (SD)	8.8 (7.3)	8.7 (6.9)	9.5 (9.0)	
Median (IQR)	7 (4-11)	7 (4-11)	7 (4-12)	

^a^information not available in 1 regional stroke audit

IS patients admitted on weekends (*p <* .0001), with an interval-onset-admission time less than 3 h (*p <* .0001), with hypertension (*p <* .0001), and hyperlipidemia (*p <* .0001) are more likely, and patients with a previous stroke (0.0055) were less likely to be admitted directly to a SU. Contrary, patients with a severe stroke (NIHSS >15) had a lower probability of being admitted to a SU (*p <* .0001) (Table 2). Results remained stable in a sensitivity analysis including all stroke subtypes, except for age. Patients younger than 85 years were more likely to be admitted on a SU (*p =* 0.0031). An additional sensitivity analysis based on all data including missing information as separate categories showed no substantial differences (data not shown).

**Table 2:** Probability for admission of patients with ischemic stroke on a Stroke Unit (demographical and clinical characteristics)

	OR (95% CI) *n* = 88,316	*P*-values
Age categories		0.0664
< 65	1	
65-74	1.03 (0.97 – 1.08)	
75-84	1.00 (0.95 – 1.05)	
≥ 85	0.95 (0.89 – 1.01)	
Sex		
Men	1	0.3088
Women	0.98 (0.94 – 1.02)	
Comorbidities		
Atrial fibrillation	1.02 (0.98 – 1.07)	0.2998
Diabetes mellitus	0.99 (0.95 – 1.03)	0.4659
Hypertension	1.13 (1.08 – 1.19)	<.0001
Previous stroke	0.94 (0.90 – 0.98)	0.0055
Hyperlipidemia	1.26 (1.21 – 1.31)	<.0001
Interval onset admission, hours^a^		
< =2	1	<.0001
> 2-3	0.94 (0.87 – 1.02)	
> 3-6	0.88 (0.83 – 0.94)	
> 6-24	0.90 (0.85 – 0.96)	
> 24-48	0.61 (0.56 – 0.66)	
> 48	0.19 (0.18 – 0.20)	
unknown	0.43 (0.41 – 0.46)	
NIHSS admission		
≤ 3	1	<.0001
≥ 4- ≤ 15	1.30 (1.25 – 1.36)	
> 15	0.55 (0.52 – 0.59)	
Day of admission^a^		
weekday	1	<.0001
weekend	1.12 (1.08 – 1.17)	

^a^no information in one regional stroke register

### Quality indicators

Results of achieved QIs by structural and individual factors are presented in Table 3. In hospitals with a SU, overall a mean of 93.7% (SD 13.3) of the QIs were within the predefined target ranges. The proportion of patients admitted to SUs (*p =* 0.0002) had a significant impact on the achievement of quality indicators, whereas year of certification (*p =* 0.3014) and number of treated patients (*p =* 0.7238) showed no significant impact. Results remained stable in a sensitivity analysis stratifying by admission on a SU (Additional file 1: Table S1).

**Table 3:** Achieved QIs by structural factors in hospitals with certified SUs^a^

	Hospitals with certified Stroke Units		Regression coefficient in % (95%-CI)^b^	*P*-values
	No of hospitals	Mean (SD) % achieved QIs		
Overall/Intercept	255	93.7 (13.3)	86.8 (79.4 – 94.3)	
SU admission within hospitals with SU, %				
< 60	16	87.6 (18.5)	0	0.0002
60-80	63	92.7 (14.3)	4.4 (1.2 – 7.5)	
> 80	176	94.4 (12.4)	5.7 (2.7 – 8.6)	
Year of certification^c^				
2000-2002	31	94.2 (12.5)	0	0.3014
2003-2005	42	93.3 (13.3)	-0.9 (-2.6 – 0.7)	
2006-2008	32	93.8 (14.2)	0.7 (-1.3 – 2.9)	
2009-2012	81	93.6 (13.3)	-0.6 (-2.1 – 0.9)	
No of patients				
< 250	11	94.4 (12.9)	0	0.7238
250-500	62	92.4 (15.1)	1.8 (-4.9 – 8.4)	
> 500-750	80	94.0 (13.1)	2.4 (-4.1 – 8.9)	
> 750	102	93.7 (13.0)	1.8 (-4.7 – 8.3)	

^a^11 quality indicators; ^b^analysis adjusted for individual level factors sex, stroke subtype, age, weekday of admission, NIHSS, ^c^no information about year of certification in one regional stroke register; unknown year of certification of hospitals occurred in every register

## Discussion

Demographic and clinical factors influencing admission to a SU were identified in a large sample of hospitals across Germany. Results of our study show that patients admitted to SUs have milder strokes, suffer more from high blood pressure or hyperlipidemia, and are admitted earlier after stroke onset. A higher proportion of quality indicators were fulfilled in hospitals with a higher proportion of patients being directly admitted to a SU.

Our findings are comparable with previous studies showing that patients treated on a SU tends to be younger on average, had a higher prevalence of comorbidities, especially atrial fibrillation, and hypertension, and were more conscious [3, 17]. Older patients might be more likely to be admitted to a geriatric or general ward, especially older patients with multiple pathologies and greater degrees of frailty [18]. We found a slightly higher probability for patients directly admitted to SUs on weekends. This might be explained by certification requirements of SUs resulting in better staffing of SUs compared to other wards on weekends which could lead to a preferred admission to a SU. Data on quality of care on weekends are heterogeneous. Most studies demonstrate a poorer quality of care, and negative effects for patients being admitted on weekends [18–21], whereas others do not confirm these findings [22–24].

Adherence to given target values of evidence-based QIs was significantly associated with the proportion of patients admitted to a SU. That means, more patients receive guideline-recommended therapy if they are treated on a SU. This implies that ischemic stroke patients should be treated on a SU. But, not all quality indicators are relevant for patients not being treated on a SU – e.g. patients with intracerebral hemorrhage treated on an Intensive Care Unit. This higher quality of stroke care might be the result of a more specific training of the medical staff of SU (doctors, nurses, and therapists) in acute stroke management. A higher proportion of quality standards met in patients admitted to a SU was also reported by Rudd et al [18]. We found no association between proportion of fulfilled QIs and year of first certification, or the annual number of stroke patients treated. We assume that the quality of stroke care could be maintained even if certification was several years ago. This finding is in contrast to other studies reporting a higher level of quality of care and better outcomes for patients in higher volume SUs [25–27]. Our results suggest that certification requirements of the DSG and the SDSH might ensure quality of SU care independently from the number of patients treated on a local SU per year. We found no association between year of certification and number of QI within given target values. There is not much comparable evidence on this subject as previous studies compared certified- and non-certified stroke centers [28, 29] or different certification procedures [30]. One strength of the present analyses is the large number of patients analyzed. Data are routinely checked for plausibility, but coding errors at hospital level cannot be ruled out. Because in Germany participation in the ADSR is mandatory only for SUs and in specific regions, we decided not to compare quality of SU care between hospitals with and without SUs, in order to avoid bias due to most an overrepresentation of hospitals with SU care in the dataset and, thus, makes comparisons with centers not providing SU care difficult

Overall from 180,887 stroke patients, 82.4% were directly admitted to a SU. There seems to be still room for quality improvement regarding the proportion of stroke patients admitted to a SU. Our data contains only information on the admission of a SU. It is possible that patients were transferred from a normal ward to a SU during hospital stay. Unfortunately, this information was not included in our dataset.

The process of admission to a SU might be affected by selection bias as severely affected patients e.g. with intracerebral or subarachnoid hemorrhage are more likely to be admitted to intensive care units or neurosurgical departments. In addition, but unlikely to affect our results relevantly, we might underestimate the entire proportion of patients being treated on a SU, while secondary admissions during the hospital stay were not recorded.

## Conclusions

Our data indicate a high quality of acute SU care in Germany according to predefined quality indicators. The influence of documented structural characteristics on adherence to evidence-based quality indicators was low. However, there might be still room for quality improvement left regarding the proportion of stroke patients admitted to a SU.

## Additional file

Additional file 1: Table S1. Achieved QIs by structural factors in hospitals with certified SUs. Additional analysis to Table 3 of the manuscript. (DOCX 50 kb)

## Abbreviations

ADSR: German Stroke Register Study Group (Arbeitsgemeinschaft Deutscher Schlaganfall Register)
DSG: German Stroke Society (Deutsche Schlaganfall Gesellschaft)
GLMM: Generalized linear mixed models
ICH: Intracerebral hemorrhage
IQR: Interquartile range
IS: Ischemic stroke
NIHSS: National Institutes of Health Stroke Scale
QI: Quality indicator
RCTs: Randomized clinical trials
SD: Standard deviation
SDSH: German Stroke Foundation (Stiftung Deutsche Schlaganfall-Hilfe)
SU: Stroke unit
TIA: Transient ischemic attack
