# Supplementary material for: Stroke unit care in germany: the german stroke registers study group (ADSR)
Source: BMC Neurol. 2017 Mar 9;17:49. doi: 10.1186/s12883-017-0819-0 (PMC5343401; doi:10.1186/s12883-017-0819-0)
Supplement: Additional file 1: Table S1. — Achieved QIs by structural factors in hospitals with certified SUs. Additional analysis to Table 3 of the manuscript. (DOCX 50 kb) [file 12883_2017_819_MOESM1_ESM.docx]

ONLINE SUPPLEMENT

Supplemental Table I Achieved QIs by structural factors in hospitals with certified SUs*

|  |  | **Admission on a SU** | | | **Admission not on a SU** | | |
| --- | --- | --- | --- | --- | --- | --- | --- |
|  | No of hospitals | Mean (SD) % achieved QIs | Regression coefficient in % (95%-CI) † | p-values | Mean (SD) % achieved QIs | Regression coefficient in % (95%-CI) † | p-values |
| Overall / Intercept | 255 | 94.8 (11.6) | 89.5 (82.3 – 96.7) |  | 88.5 (18.0) | 83.0 (71.3 – 94.7) |  |
|  |  |  |  |  |  |  |  |
| SU admission within hospitals with SU, % |  |  |  | 0.0228 |  |  | 0.0419 |
| <60 | 16 | 90.9 (14.9) | 0 |  | 84.4 (21.0) | 0 |  |
| 60-80 | 63 | 94.2 (12.5) | 3.6 (0.5 – 6.6) |  | 88.8 (17.6) | 2.9 (-1.7 – 7.6) |  |
| >80 | 176 | 95.2 (11.1) | 4.0 (1.1 – 6.9) |  | 88.7 (17.2) | 4.6 (0.1 – 9.1) |  |
|  |  |  |  |  |  |  |  |
| Year of certification‡ |  |  |  | 0.4055 |  |  | 0.5798 |
| 2000-2002 | 31 | 95.0 (11.3) | 0 |  | 90.6 (16,7) | 0 |  |
| 2003-2005 | 42 | 94.4 (11.8) | -0.6 (-2.2 – 1.0) |  | 88.8 (17,3) | -1.7 (-4.3 – 0.9) |  |
| 2006-2008 | 32 | 95.1 (12.1) | 1.0 (-1.0 – 3.0) |  | 87.3 (20,6) | -0.7 (-3.9 – 2.5) |  |
| 2009-2012 | 81 | 94.9 (11.5) | -0.3 (-1.8 – 1.1) |  | 87.8 (18,3) | -1.3 (-3.7 – 1.1) |  |
|  |  |  |  |  |  |  |  |
| No of patients |  |  |  |  |  |  |  |
| <250 | 11 | 95.6 (11.4) | 0 | 0.7028 | 86.7 (18,2) | 0 | 0.9840 |
| 250-500 | 62 | 94.1 (12.6) | 1.6 (-4.8 – 8.0) |  | 85.6 (20,9) | 2.0 (-8.5 – 12.6) |  |
| >500-750 | 80 | 95.1 (11.5) | 2.3 (-4.0 – 8.5) |  | 88.1 (18.6) | 2.1 (-8.3 – 12.4) |  |
| >750 | 102 | 94.8 (11.5) | 1.7 (-4.6 – 7.9) |  | 89.2 (17.3) | 2.1 (-8.2 – 12.4) |  |

*11 quality indicators; †analysis adjusted for individual level factors sex, stroke subtype, age, weekday of admission, NIHSS, ‡ no information about year of certification in one regional stroke register; unknown year of certification of hospitals occurred in every register
